# Wake EEG and Sleep Hypoxemia Predicts Poor Driving and Vigilance Following Extended Wakefulness in People With OSA

**DOI:** 10.1111/jsr.70131

**Published:** 2025-07-09

**Authors:** Andrew Vakulin, Garry Cho, David Stevens, Nathaniel S. Marshall, Hannah Openshaw, Delwyn J. Bartlett, Caroline D. Rae, Keith K. H. Wong, R. Doug McEvoy, Ronald R. Grunstein, Angela L. D'Rozario

**Affiliations:** ^1^ Flinders Health and Medical Research Institute, Sleep Health/Adelaide Institute for Sleep Health, College of Medicine and Public Health Flinders University Adelaide Australia; ^2^ Sleep and Circadian Research Group (CIRUS) Woolcock Institute of Medical Research Sydney Australia; ^3^ Centre for Nutrition and Gastrointestinal Diseases South Australian Health & Medical Research Institute Adelaide Australia; ^4^ Faculty of Medicine and Health University of Sydney Sydney Australia; ^5^ Neuroscience Research Australia Sydney Australia; ^6^ School of Psychology The University of New South Wales Sydney Australia; ^7^ Royal Prince Alfred Hospital, and Sydney Health Partners Sydney Australia; ^8^ School of Psychology, Faculty of Science, Brain and Mind Centre and Charles Perkins Centre University of Sydney Sydney Australia

**Keywords:** alertness, alertness failure, EEG slowing, power spectral analysis, sleep‐disordered breathing

## Abstract

Obstructive sleep apnoea (OSA) is a highly prevalent but heterogeneous condition which makes identifying patients at risk of vigilance and driving impairment clinically challenging. Resting wake electroencephalography (EEG) is associated with vigilance performance in healthy participants. We examined if rested wake EEG predicted vigilance and driving impairment in OSA following extended wakefulness. Fifty‐four patients underwent baseline polysomnography and 28‐h extended wakefulness, repeated vigilance assessments (psychomotor vigilance task (PVT), driving simulator) and resting wake EEG (Karolinska drowsiness test). Cluster analysis assigned patients into groups of either resistant (*n* = 38) or vulnerable (*n* = 16) to vigilance failure based on PVT and driving performance following extended wakefulness. Backward stepwise regression models and receiver operator characteristics curves were used to determine the strongest clinical and wake EEG predictors of vigilance impairment. The vulnerable OSA group showed impaired PVT and driving performance relative to the resistant group (*p* < 0.01). Compared with resistant patients, the vulnerable group exhibited increased delta (*p* < 0.001) and theta (*p* = 0.003) EEG power across extended wake. Significant predictors of vigilance impairment were (1) baseline wake EEG theta and O_2_ Nadir during sleep, explaining 42% of the variance, and (2) delta power and O_2_ Nadir explaining 32% of the variance in vigilance performance. ROC analysis showed strong discrimination between vulnerable and resistant patients (AUC 0.85–0.86, sensitivity 73%–87%, specificity 71%–84%). Slow frequency wake EEG activity and sleep hypoxemia at baseline are predictive of subsequent driving simulator and vigilance impairment in patients with OSA following extended wakefulness. This is potentially important for vigilance and fitness‐to‐drive assessments in OSA.

**Trial Registration:** Australian New Zealand Clinical Trials Registry: ACTRN12613001171707.

## 1 Introduction

Obstructive sleep apnoea (OSA) is highly prevalent globally (Benjafield et al. 2019; Lechat et al. 2022) and is associated with excessive daytime sleepiness (EDS) and fragmented sleep, impaired alertness and an increased risk of impaired driving performance, motor vehicle accidents (MVA) and workplace accidents (Lindberg et al. 2001; Tregear et al. 2009).

In healthy participants, there is high inter‐individual variability in alertness and neurobehavioral function following sleep loss (Tkachenko and Dinges 2018). This variability is greater in patients with OSA, driven by a small ‘vulnerable’ sub‐group of patients at higher risk of alertness failure following sleep loss (Vakulin et al. 2014). Importantly, standard clinical measures of OSA severity such as the apnoea hypopnea index (AHI), hypoxemia or subjective EDS do not consistently predict impaired alertness in OSA patients under baseline or extended wakefulness conditions (D'Rozario et al. 2017; Vakulin et al. 2016). This limits their clinical utility to identify patients at high risk of alertness failure during fitness to drive/work assessments, highlighting the need for better clinically deployable assessment tools (Vakulin et al. 2012).

In healthy participants, wake electroencephalography (EEG) theta and delta power have been shown to progressively increase with prolonged time awake (Hung et al. 2013; Chua et al. 2014; Finelli et al. 2000; Cajochen et al. 1999) and correlate with subjective sleepiness (Drapeau and Carrier 2004) and neurobehavioral function (Hung et al. 2013; Chua et al. 2014; Cajochen et al. 1999; Torsvall and Akerstedt 1987), suggesting wake EEG might be a marker of state alertness and performance. For example, wake EEG theta power is elevated in participants who are vulnerable to performance impairment on the psychomotor vigilance task (PVT) following 24 h of extended wakefulness (Chua et al. 2014).

Previous research in OSA has demonstrated the potential for overnight sleep EEG power spectra to predict neurobehavioral impairment, including vigilance and driving performance (D'Rozario et al. 2017; Vakulin et al. 2016; Mullins et al. 2020). However, a brief awake EEG assessment to ascertain OSA patient risk of alertness failure and accident risk would be clinically advantageous due to the relative brevity of the assessment (~10 min) compared to overnight sleep study EEG. However, the relationship between wake EEG and neurobehavioral function in OSA is currently uncertain. Only two small studies observed a relationship between wake EEG and future driving simulator performance impairment (D'Rozario et al. 2013) or concurrent subjective sleepiness (Greneche et al. 2008) in OSA during extended wakefulness. D'Rozario et al. (2013) found that a novel quantitative EEG analysis method (detrended fluctuation analysis) in eight OSA patients from their wake EEG at baseline correlated with driving simulator impairment following 24 h of extended wakefulness, while traditional EEG power spectra did not. An older study by Greneche et al. (2008) reported on 12 OSA patients and 8 controls studied during 24 h of sustained wakefulness and found a relationship between subjective sleepiness ratings and wake EEG theta, alpha and beta power in the controls, but not in the OSA patients. This study did not evaluate objective vigilance or driving performance. Hence, there remains significant uncertainty regarding the relationship between wake EEG and vigilance or driving performance impairment as a result of sleep loss.

Therefore, the aims of this study were to determine if wake EEG markers measured at rested baseline can discriminate between patients with OSA who are found to be vulnerable or resistant to vigilance and driving impairment following extended wakefulness. We hypothesised that baseline wake EEG theta power (4.5‐8 Hz) would be higher in OSA patients who are vulnerable to alertness failure measured by driving simulator and psychomotor vigilance impairment following extended wakefulness.

## 2 Methods

The study was approved by the Sydney Local Health District (RPAH Zone) protocol No. X12‐00828 and HREC/12/RPAH/40. All participants were reimbursed AUD370 for their participation. This was a sub‐study of a larger trial (ACTRN12613001171707).

### 2.1 Participant Recruitment and Screening

**FIGURE 1:**
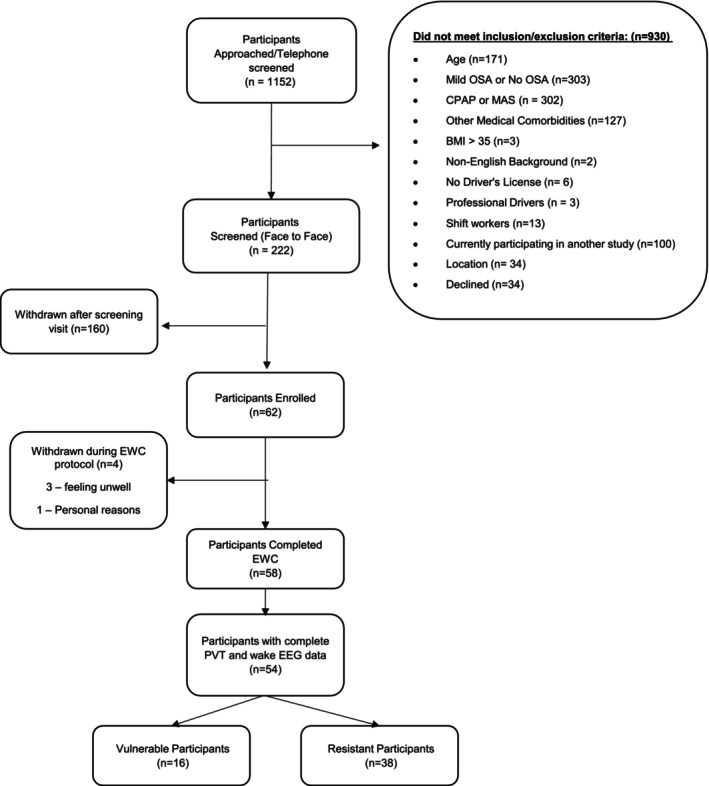
Consort diagram of participant screening and recruitment.

A summary of participant recruitment is shown in Figure 1. Participants were recruited from sleep clinics at the Woolcock Institute of Medical Research and the Royal Prince Alfred Hospital, Sydney, Australia, or via media streams (television, newspaper). *Inclusion criteria*: Males and females; age 25–70; polysomnography (PSG) confirmed OSA (apnoea hypopnea index, AHI > 10 events/h), or oxygen desaturation index (ODI ≥ 3%) > 8 events/h using 2007 American Academy of Sleep Medicine criteria (Iber et al. 2007); had not been previously treated for OSA for > 3 months or had been off any treatment for at least 6 months, hold a private driver's licence and drive regularly (≥ 2 h/week). *Exclusion criteria*: Evidence of other sleep disorders (idiopathic hypersomnia, narcolepsy, restless legs), clinically significant co‐morbidity; uncontrolled medical conditions e.g., cardiac failure, hypertension, hypercapnia; history of head injury or psychiatric/neurological disorder (including stroke); use of central nervous system active agents (sedative hypnotics; opiates, recreational drugs), heavy alcohol consumption (> 40 g daily); current shift‐worker (due to instability in sleep/wake behaviour); professional drivers (as this study targeted private drivers).

### 2.2 Introductory Visit and Pre‐Laboratory Assessment

During the introductory visit, informed consent was obtained and all participants completed health and sleep questionnaires including the Pittsburgh Sleep Quality Index (PSQI) (Buysse et al. 1989), Epworth Sleepiness Scale (ESS) (Johns 1991), Insomnia Severity Index (ISI) (Bastien et al. 2001), Depression, Anxiety and Stress Scale (DASS) (Brown et al. 1997) and the state component of the State–Trait Anxiety Inventory (Spielberger et al. 1970), the Morningness–Eveningness Questionnaire (Horne and Ostberg 1976) and driving history. Participants completed a short practice version of all cognitive tests and driving simulation (30 min drive) to become familiar with the procedures, minimise any learning effects and to screen for simulator sickness.

Prior to the experimental visit, participants wore an actigraph (Actiwatch 2, Philips Respironics, Oregon USA) and completed a sleep diary for 5–7 days prior to the extended wakefulness visit to assess habitual sleep and wake habits.

### 2.3 Extended Wakefulness Protocol

**FIGURE 2:**
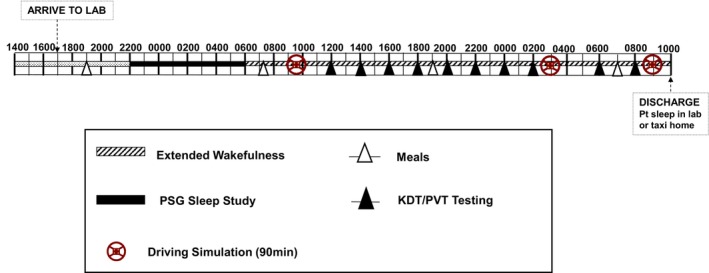
Protocol timeline of the experimental extended wakefulness protocol, including timing of baseline polysomnography, driving simulator testing and repeated cognitive/PVT testing.

Participants attended the sleep laboratory at 5 pm, were provided dinner and set up for a full PSG (sleep opportunity 22:00–06:00) followed by 28 h extended wakefulness (EW) (Figure 2). Lighting was kept at < 1 lx during sleep and 40–50 lx during wakefulness and room temperature was kept between 22°C–24°C. Food consumption time and caloric intake (2500–3000 kJ/meal) were standardised. During the EW, the PVT and Karolinska drowsiness test (KDT) were conducted every 2 h (10 times in total). Following 6.00 am wake‐up time and prior to the first driving simulator test (9.00 am–10.30 am) and cognitive testing battery (PVT and KDT) at midday, all participants were allowed to shower, had EEG and ECG reapplied, and had an opportunity to have breakfast. Participants were monitored by staff visually at all times to ensure there was no possibility of naps prior to driving and cognitive assessments.

### 2.4 Sleep and Respiratory Measurements

PSG was performed using Embla Titanium acquisition hardware (Embla Systems, Colorado, USA). The EEG montage included CPz as reference, at scalp positions F3‐M2, Fz‐(average M1 + M2), F4‐M1, C3‐M2, Cz‐(average M1 + M2), C4‐M1, Pz‐(average M1 + M2), O1‐M2, Oz‐(average M1 + M2), O2‐M2; left and right electrooculogram, submental electromyogram, plantar flexor EMG, nasal cannula and thermocouple to measure airflow, inductive plethysmography for thoraco‐abdominal motion, lead II electrocardiography and finger pulse oximetry. EEG signals were digitised with a 512 Hz sampling rate, a high‐pass filter at 0.3 Hz, and a low‐pass filter at 50 Hz. Sleep and respiratory scoring was performed according to the 2007 American Academy of Sleep Medicine criteria (Iber et al. 2007). Apneas were defined as cessations of nasal flow lasting ≥ 10 s. Hypopneas were defined as a > 50% decrease in nasal flow (or in both thoracic and abdominal excursions) and associated with either a ≥ 3% oxygen desaturation or an EEG arousal.

### 2.5 Karolinska Drowsiness Test (KDT)

The KDT is a 7.5‐min wake EEG recording where participants are instructed to remain seated whilst focusing on a target on the wall with their eyes open (2.5 min), then eyes closed (2.5 min), then eyes open (2.5 min).

### 2.6 Psychomotor Vigilance Task (PVT)

The 10‐min PVT was conducted using a handheld device (PVT‐192, Ambulatory Monitoring Inc., New York, USA). Participants are required to react to approximately 98–100 visual stimuli (LED screen counter) and respond by pressing a button as quickly as possible. Each consecutive stimulus was presented at an inter‐stimulus interval of 2000–10 000 milliseconds. The key PVT variables utilised in the current analysis were the reciprocal reaction time (RRT) and PVT lapses (RT > 500 ms) as these metrics are reported to have the highest effect size and sensitivity to sleep loss in total and partial sleep deprivation studies (Basner and Dinges 2011).

### 2.7 AusEd Driving Simulator Assessment

The AusEd driving simulator task (Woolcock Institute of Medical Research, Sydney, Australia) (Desai et al. 2007) simulates driving on a country road at night. The driving task involved a 90‐min monotonous country scenario as described previously (Vakulin et al. 2014, 2021). The driving test was administered three times during the EW protocol: at baseline 9.00 am–10.30 am (3 h of wakefulness) and following EW 2.30 am–4.00 am (22.5 h of wakefulness, coinciding with circadian nadir) and 8.00 am–9.30 am the following morning (26.5 h of wakefulness). The main outcome measures were average steering deviation and the number of simulator crash events from the second driving test following 22.5 h of wakefulness (see Supporting Information and Figure S1 for more information).

### 2.8 Data Processing and Statistical Analysis

Quantitative EEG (qEEG) analysis used an internally developed algorithm to process artefact and calculate EEG spectral power, described previously (D'Rozario et al. 2013; Kim et al. 2009). EOG artefact was processed using independent component analysis (ICA) and EEG signals were manually reviewed by a trained researcher. The resting wake EEG recording was segmented as follows: eyes open 1: start of the recording to 2 min, eyes closed 2:30 to 4:30, eyes open 2: 5:00–7:00 (eyes open). Thirty‐second segments between the eyes open and eyes closed conditions were discarded from analysis due to behavioural change in response to instructions (eye closure and opening). KDT recordings from each participant were quantitatively analysed using Cz‐ (M1 + M2)/2) EEG derivation. Absolute qEEG power was calculated across frequency ranges of 0.5–4.5 Hz (delta), 4.5–8 Hz (theta), 8–12 Hz (alpha) and 12–32 Hz (beta) for eyes closed and open conditions.

## 3 Defining Vulnerability Groups

A two‐step hierarchical cluster analysis was used to group OSA patients into vulnerable (poor performance) or resistant groups (good performance) based on average PVT and driving simulator performance following EW. Two‐step cluster analysis is an approach that identifies homogeneous groups of cases based on the distribution of the input variables (Kent et al. 2014). Four data variables were inputs into the cluster analysis, including two PVT variables: averaged RRT and lapses from 3 consecutive PVT tests (18, 20, 24 h of wakefulness) and two driving simulator variables: steering deviation and number of crashes from the second driving test (22.5 h of wakefulness). The cluster analysis was not constrained and automatically determined the cluster membership based on the distribution of the four data inputs (see Figure S1).

All statistical analysis was performed using IBM SPSS Statistics 23 software (IBM, New York, USA). Log (Hung et al. 2013) transformation was applied to not normally distributed data. Pearson's correlations examined univariate associations between baseline clinical, demographic and PSG measures with continuous PVT and driving simulator outcomes following EW (average of three PVT tests at 18, 20 and 24 h of wake and driving simulator test at 22.5 h of wake).

**FIGURE 3:**
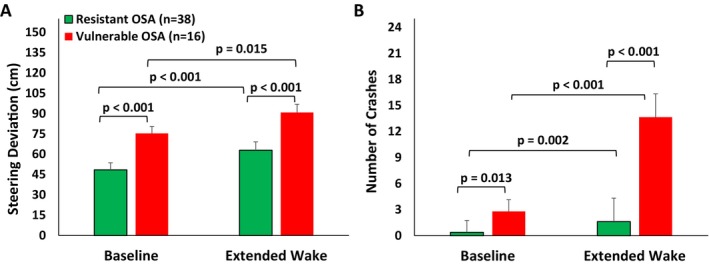
AusEd driving simulator steering deviation (A) and crash data (B) at baseline and following extended wakefulness in resistant and vulnerable OSA groups. Paired and unpaired samples *t*‐tests were used to examine the mean differences in driving simulator steering deviation performance (Figure 3A) between the vulnerable and resistant OSA groups and baseline vs. extended wakefulness. Mann–Whitney‐U test was used to compare the number of driving simulator crashes between the vulnerable and resistant groups and Wilcoxon Signed‐Rank test was used to compare baseline vs. extended wake condition. Data presented as mean ± SEM.

Unpaired samples *t*‐tests examined mean differences in anthropometrics, PSG metrics, questionnaires (Table 1) and driving simulator steering deviation (Figure 3A) between vulnerable and resistant OSA groups. Mann–Whitney U test compared the number of driving simulator crashes and Wilcoxon Signed‐Rank test compared baseline vs. extended wake conditions (Figure 3B). Linear mixed models compared PVT performance and EEG power between groups (vulnerable vs. resistant OSA) and time (10 testing sessions), with subject as a random factor and autoregressive (AR1) covariance structure to control for serial correlation between adjacent observations.

**TABLE 1:** Anthropometric, PSG and Questionnaire Variable in all OSA patients and split by vulnerability groups.

Variable	All OSA	Resistant OSA	Vulnerable OSA	Group Comparison *p*
*N*	54	38	16	
Gender (M/F)	46/8	33/5	13/3	
Age (yr)	51.5 [43.0 to 55.0]	50.5 [42.0 to 54.0]	53.5 [45.5 to 57.0]	0.165
Body mass index^a^	31.0 [28.2 to 34.7]	31.0 [28.8 to 33.0]	34.0 [27.0 to 42.3]	0.063
Epworth sleepiness score	10.0 [7.0 to 12.8]	9.0 [6.3 to 11.0]	11.5 [9.3 to 14.0]	0.073
Sleep onset latency (min)^a^	4.3 [1.4 to 9.1]^b^, ^e^	4.8 [1.7 to 10.0]	3.4 [1.1 to 4.7]	0.145
Wake after sleep onset (min)	46.3 [29.8 to 64.5]	55.5 [37.6 to 65.8]	29.6 [25.0 to 48.6]	0.026
Total sleep time from PSG (min)	414.5 [380.3 to 432.0]^b^, ^c^	398.9 [373.4 to 426.9]	424.3 [413.1 to 446.8]	0.013
Total sleep time from actigraphy (min)	386.5 [341.5 to 437.5]^e^	401.0 [343.5 to 442.0]	351.5 [332.5 to 405.5]	0.212
Sleep efficiency (%)	88.9 [83.1 to 92.2]	86.3 [81.7 to 90.8]	92.2 [88.5 to 94.4]	0.013
% Stage N1 sleep	3.7 [2.4 to 6.3]^b^	4.5 [2.6 to 6.6]	3.3 [2.4 to 4.0]	0.090
% Stage N2 sleep	59.2 [52.8 to 66.5]	57.2 [52.2 to 64.6]	61.0 [59.2 to 75.6]	0.205
% Stage N3 sleep	16.4 [11.1 to 21.7]	16.2 [11.7 to 21.4]	16.5 [8.2 to 21.8]	0.725
% REM sleep	17.9 [14.8 to 22.7]	18.5 [14.9 to 23.1]	15.6 [14.5 to 20.2]	0.140
Apnoea hypopnea index (events/h)^a^	31.6 [18.4 to 52.4]^e^	28.0 [15.2 to 47.1]	50.9 [28.0 to 63.7]	0.014
Oxygen desaturation index (events/h)^a^	23.7 [12.4 to 43.0]^e^	18.0 [8.8 to 32.8]	43.9 [24.8 to 57.9]	0.003
Arousal index (events/h)^a^	24.4 [16.0 to 43.3]	23.2 [17.4 to 35.2]	40.3 [14.8 to 50.3]	0.125
% time < 90% SaO2^a^	0.4 [0.0 to 2.6]	0.2 [0.0 to 2.0]	0.9 [0.1 to 9.8]	0.104
Average oxygen desaturation (%)^a^	4.9 [4.1 to 5.6]^b^, ^c^, ^d^, ^e^	4.7 [3.8 to 5.3]	5.1 [4.4 to 8.2]	0.031
Oxygen saturation nadir (%)^a^	82.0 [78.0 to 86.0]^b^, ^c^, ^d^, ^e^	84.0 [80.0 to 87.0]	76.5 [63.5 to 81.3]	0.003
Morningness eveningness questionnaire	28.0 [25.0 to 31.0]	28.5 [25.0 to 31.8]	26.0 [24.5 to 30.3]	0.796
Depression score (DASS‐depression)^a^	4.0 [2.0 to 8.0]	2.0 [0.5 to 8.0]	6.0 [2.0 to 10.0]	0.193
Anxiety score (DASS‐anxiety)^a^	2.0 [2.0 to 6.0]	2.0 [0.0 to 4.0]	3.0 [2.0 to 11.0]	0.110
Stress score (DASS‐stress)^a^	7.0 [2.5 to 13.5]	6.0 [0.5 to 12.0]	9.0 [5.5 to 14.5]	0.166
Pittsburgh sleep quality index	7.0 [5.0 to 11.5]	6.5 [5.0 to 9.0]	7.5 [5.8 to 13.0]	0.095
Insomnia severity index	10.5 [8.0 to 14.8]	10.0 [7.3 to 13.0]	12.0 [9.0 to 18.3]	0.242
Trait anxiety score (STAI‐trait)	43.5 [42.0 to 47.0]	43.0 [41.3 to 47.0]	45.0 [42.0 to 48.0]	0.206

*Note*: Values are Medians and Inter quartile ranges, Total Sleep Time from Actigraphy is the average over a 5–7 day/night actigraphy monitoring, Total Sleep Time from PSG is from single baseline night during laboratory extended wakefulness visit.

^a^ Indicates variables with abnormal distribution.

^b^ Indicates significant bivariate correlation with PVT Lapses following extended wakefulness—*p* < 0.01.

^c^ Indicates significant bivariate correlation with PVT Reciprocal Reaction Time following extended wakefulness—*p* < 0.01.

^d^ Indicates significant bivariate correlation with driving simulator steering deviation following extended wakefulness.

^e^ Indicates significant bivariate correlation with driving simulator crashes following extended wakefulness. Group Comparison *p*‐values represents the comparison between the Resistant vs Vulnerable OSA groups with unpaired *t*‐test *p*‐values shown for normally distributed variables and Mann–Whitney U test shown for non‐normally distributed variables.

Backward stepwise multiple linear regression models were used to examine which baseline demographic, PSG and baseline wake EEG variables were significant predictors of continuous PVT and driving simulator performance following EW. To define the baseline wake EEG predictors, we averaged the 3 consecutive baseline KDT tests at 8, 10 and 12 h awake (Figure 2) and were considered a good representation of wake EEG during the baseline period, covering the afternoon nadir and the wake maintenance zone prior to significant sleep deprivation following 16 h awake. We have also performed sensitivity analysis by utilising the wake EEG data from each of the three KDT tests individually instead of the average wake EEG data from the three consecutive KDT tests. The very first KDT test at midday (6 h awake) was not utilised to define baseline wake EEG due to 25% of participants missing this test battery due to protocol timing delays. For this analysis, the three PVT and second driving performance variables from the EW period were reduced to a single Vigilance Performance Factor using principal component factor analysis (see Supporting Information for details).

Finally, receiver operating characteristic (ROC) curve analysis examined if significant predictor metrics from the regression models discriminated between vulnerable vs. resistant OSA patients. We used the linear regression equations derived from the combination of predictor variables as a continuous test variable and the vulnerable versus resistant group status as the binary classification outcome. All data are presented as mean ± SD or median ± IQR and *p* < 0.05 was considered statistically significant unless otherwise specified.

## 4 Results

Sixty‐two participants were enrolled in the study. Four participants withdrew from the study (one no reason provided, three feeling unwell [two of which were due to simulator sickness]). The final analyses included 54 participants with complete PVT, driving, and wake EEG data (Figure 1). There were significant univariate associations between several baseline clinical and PSG variables with PVT and driving performance following EW, including total sleep time, sleep onset latency and hypoxemia metrics (see Table 1, univariate correlations indicated by symbols).

**FIGURE 4:**
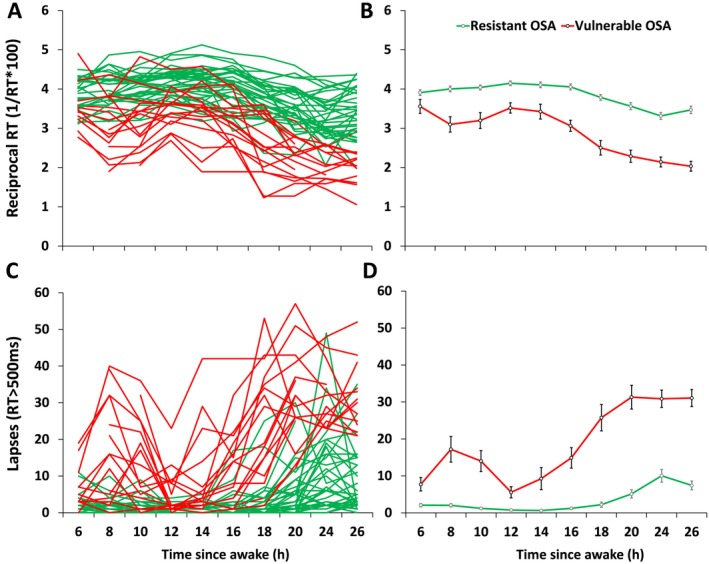
PVT individual level data for reciprocal reaction time (A) and lapses (C), and mean ± SEM data for reciprocal reaction time (B) and lapses (D), split by vulnerable and resistant OSA groups across all 10 PVT tests during the 26 h extended wakefulness protocol.

Cluster analysis successfully classified all patients with OSA into either a resistant (*n* = 38) or a vulnerable group (*n* = 16) (Figures 3 and 4) (Figures S2 and S3). There was a significant main effect of group, time and a group × time interaction (all *p* < 0.001) for both PVT RRT and lapses (Figure 4). The vulnerable patients had significantly slower reaction time and more lapses compared with the resistant patients across EW (all *p* < 0.01). The vulnerable patients also exhibited pronounced circadian variation across the 24‐h testing period relative to the resistant patients. There was also a significant mean difference between the vulnerable and resistant groups in driving simulator steering deviation (baseline and EW) and crash events (EW) (Figure 3).

The anthropometric, PSG and questionnaire data are shown in Table 1. The vulnerable OSA group had longer total sleep duration, shorter time spent awake and higher sleep efficiency, higher AHI and ODI and lower O_2_ Nadir during the baseline PSG than the resistant group. There were no between‐group differences in age, questionnaires or self‐reported driving distance per year (Median [IQR], resistant 3.0 [2.0–5.0] vs. vulnerable 2.0 [2.0–4.0] kilometres per annum).

### 4.1 Wake EEG Differences Between Vulnerable and Resistant OSA Patients

**FIGURE 5:**
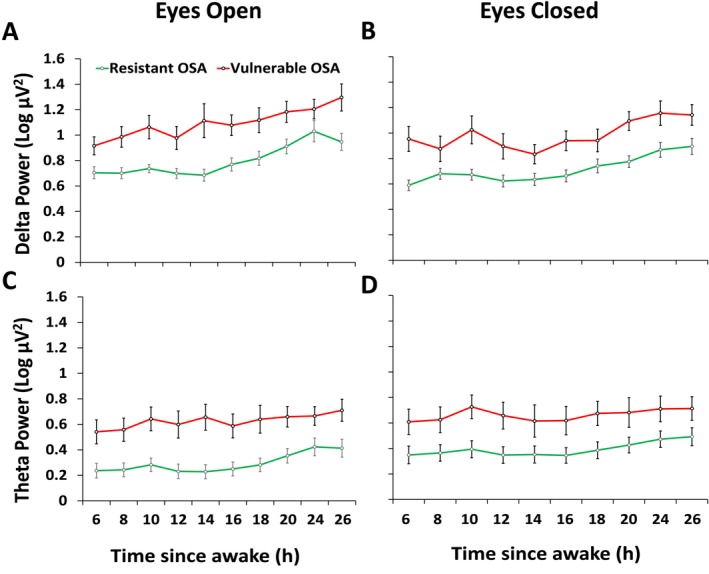
Wake EEG power in the vulnerable and resistant OSA patients across each of the 10 KDT tests during the extended wakefulness protocol. Wake EEG power is shown separately for the eyes open condition during each KDT test (left panes graph A, C, E and G) and eyes closed condition during each KDT test (right panels graphs B, D, F and H).

Figure 5A–H shows wake EEG power results for delta, theta, alpha and beta frequencies for the 10 KDT tests during EW. Delta power progressively increased in both groups throughout EW, but the vulnerable patients had elevated delta power across EW. There was a significant main effect of group (F_(1,51)_ = 13.9, *p* < 0.001) and time (F_(9,230)_ = 7.1, *p* < 0.001) for wake EEG delta power during the eyes open condition and a main effect of group (F_(1,52)_ = 11.3, *p* = 0.001) and time (F_(9,237)_ = 8.1, *p* < 0.001) for the eyes closed condition, but no interactions (Figure 5A,B).

Theta power increased over time in both groups, but the vulnerable patients had elevated theta power compared with the resistant patients throughout EW. There was a significant main effect of group (F_(1,52)_ = 9.6, *p* = 0.003) and time (F_(9,234)_ = 4.7, *p* < 0.001) during the eyes open condition and a main effect of group (F_(1,52)_ = 5.1, *p* = 0.029) and time (F_(9,229)_ = 3.0, *p* = 0.002) during the eyes closed condition, but no interactions (Figure 5C,D). There were no significant effects for other EEG frequencies.

### 4.2 Baseline Predictors of Vigilance Performance Following Extended Wakefulness

Backward stepwise linear regression results are shown in Table 2. O_2_ Nadir from the baseline PSG and wake EEG theta power from the eyes open KDT condition were significant predictors of vigilance performance following EW, explaining 42% of the variance (14% and 28%, respectively). O_2_ Nadir and wake EEG delta power were also significant predictors of vigilance performance following EW, explaining 32% of the variance (9% and 23%, respectively). Sensitivity analysis replacing the primary EEG theta and delta variables from the eyes open condition (averaged across three consecutive baseline testing sessions at 8, 10 and 12 h of wakefulness) with EEG outcomes from each of the three individual baseline tests revealed consistent findings with the primary analysis. Further sensitivity analysis to explore different metrics of hypoxemia and separately including ODI or average oxygen desaturation in the models also showed consistency with the primary analysis, where O_2_ Nadir remained a significant predictor. Taking the wake EEG theta and delta power from the eyes closed KDT conditions showed consistent findings, where only the wake EEG theta and delta outcomes and O_2_ Nadir were significant predictors, although the eyes closed models explained less variance in the vigilance performance outcomes (eyes closed EEG theta model 33% and eyes closed EEG delta model 31%).

**TABLE 2:** Backward stepwise linear regression models showing baseline clinical and wake EEG theta and delta power predictors of Vigilance Performance Factor.

Vigilance performance factor	Unstandardized *β*	Std. error	*p*	95% CI for *β*	Variance explained	Adjusted *R* ^2^
Wake EEG Theta regression model						
O_2_ Nadir	−7.6	2.2	< 0.001	−11.9 to −3.3	0.14	0.42
Wake EEG Theta (eyes open)	1.2	0.3	< 0.001	0.6 to 1.8	0.28	
Wake EEG Delta regression model						
O_2_ Nadir	−9.4	2.1	< 0.001	−13.7 to −5.1	0.23	0.32
Wake EEG Delta (eyes open)	0.9	0.30	0.006	0.3 to 1.5	0.09	

*Note*: All regression models included Age, BMI, ESS, sleep onset latency, total sleep time, AHI, O_2_ Nadir and separately wake EEG theta and delta power (eyes open).

### 4.3 Identifying Vulnerable and Resistant OSA Patients

**FIGURE 6:**
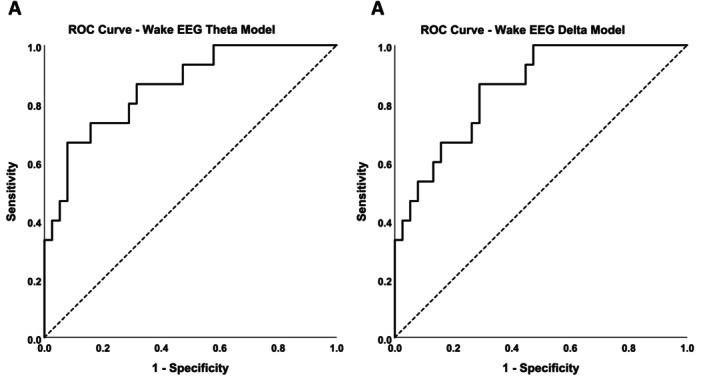
ROC analysis showing the ability of the regression equation for standardised predicted values from the wake EEG Theta model (A) and wake EEG Delta model to discriminate between vulnerable (*n* = 15) and resistant (*n* = 39) OSA patients.

The ROC analysis results are shown in Figure 6. Given the stronger models involving EEG theta and delta predictors from the eyes open relative to eyes closed condition, the ROC analysis presents findings from the eyes open conditions only. The linear combination of two baseline predictor variables (O_2_ Nadir and wake EEG theta) discriminated between vulnerable and resistant groups (Figure 6A, ROC Area Under the Curve, AUC, 0.86, 95% CI 0.73–0.99, *p* < 0.001, sensitivity 0.73 and specificity 0.84). Similarly, the linear combination of O_2_ Nadir and wake EEG delta discriminated between vulnerable and resistant groups (Figure 6B, AUC, 0.85, 95% CI 0.72–0.99, *p* < 0.001, sensitivity 0.87, specificity 0.71).

## 5 Discussion

We have shown a relationship between baseline wake EEG power and vigilance performance (PVT and driving simulator performance) following extended wakefulness in patients with OSA. Baseline slow frequency EEG delta and theta were elevated in OSA patients vulnerable to performance impairment following extended wakefulness, supporting our hypothesis. Importantly, our findings suggest that using data easily deployable in all sleep labs, wake EEG theta and delta power, when combined with hypoxemia measures, discriminate between patients who exhibit vulnerability to vigilance failure following extended wakefulness. However, excessive daytime sleepiness (ESS), the AHI and sleep fragmentation (arousals) typically also considered by clinicians to evaluate driving risk were not useful predictors. These findings provide evidence that resting wake EEG measures in the laboratory setting are potentially useful clinical markers to identify the OSA phenotype at risk of impairments in vigilance and simulated driving.

Research in healthy participants suggests wake EEG theta is a marker of homeostatic ‘sleep pressure’, with a progressive increase in theta and delta power throughout wakefulness (Hung et al. 2013; Chua et al. 2014; Finelli et al. 2000; Cajochen et al. 1999). Furthermore, wake EEG has been associated with cognitive performance during extended wakefulness highlighting the potential utility to identify cognitive impairment (Hung et al. 2013).

The design of our study resembles the study by Chua et al. (2014) who examined wake EEG in 30 healthy participants characterised as vulnerable (*n* = 15) and resilient (*n* = 15) to performance impairment on the PVT assessed every 2‐h during 26‐h extended wakefulness. Delta power was not different between the two groups at baseline. Conversely, wake theta power was significantly elevated in the vulnerable group at baseline and throughout extended wakefulness, suggesting wake theta power at baseline differentiated between vulnerable and resistant participants. In our study of OSA patients, we also observed increased wake theta power throughout the extended wake period but, in addition, we found delta power also has a similar pattern in the vulnerable OSA patients. This finding suggests that vulnerable patients, unlike vulnerable healthy participants, might exhibit higher homeostatic sleep drive and possibly disrupted slow wave activity dissipation during sleep (Finelli et al. 2000). This may also partially explain a somewhat counterintuitive observation of seemingly better quality sleep (lower WASO, greater TST and higher sleep efficiency) in the vulnerable compared with the resistant OSA group. This suggests the vulnerable group experience higher sleep drive and sleep propensity, and some evidence for this has been previously reported by our group (Cori et al. 2021) and others (Maire et al. 2014).

A previous study by D'Rozario et al. (2013) found correlations between wake EEG power (delta, theta and alpha) with subjective sleepiness, driving simulator, and PVT performance measured concurrently in a sample of eight OSA patients and nine controls during 40‐h extended wakefulness. Detrended fluctuation analysis of baseline wake EEG was correlated with simulated driving impairment after extended wakefulness, but this was not seen for power spectral EEG metrics (D'Rozario et al. 2013). Another study (Greneche et al. 2008) of 12 OSA patients and eight controls found wake EEG power in low (0.5–7.8 Hz) and fast (12.7–29.2 Hz) frequencies correlated with subjective sleepiness during 24‐h of wakefulness, but this was only in controls and the study (Greneche et al. 2008) did not assess objective performance or whether baseline wake EEG predicts subsequent impairment, making it difficult to compare the findings.

We found that wake EEG theta and delta power and sleep hypoxemia (O_2_ Nadir) were consistent predictors of vigilance performance (PVT and driving simulator) following extended wakefulness, with ROC analysis confirming these variables differentiated vulnerability vs. resistant OSA patients with moderate to high sensitivity (0.71–0.73) and specificity (0.84–0.87). Although clearly there is room to improve, these are clinically meaningful and important findings when assessing driving risk in patients with OSA, especially considering the time‐consuming nature of the standard daytime sleepiness test, the Maintenance of Wakefulness Test (MWT). Importantly, while the MWT is the recommended test for EDS and fitness to drive (Bonsignore et al. 2021), and recent evidence suggests a correlation with self‐reported accidents and near misses (Philip et al. 2021) it's relationship with driving outcomes is somewhat inconsistent and modest‐moderate at best (rho = 0.34–0.67) (Sagaspe et al. 2007; Pizza et al. 2009; Philip et al. 2008).

Lack of a healthy non‐OSA group may be considered as a limitation. However, the main objective of the current study was not to compare EEG and vigilance performance between patients and controls, which is well established by our group (Vakulin et al. 2014, 2009; D'Rozario et al. 2017, 2013) and others (Greneche et al. 2008). Rather, our aim was to determine if baseline wake EEG measures differ between clinical patients identified as vulnerable or resistant to vigilance impairment following extended wakefulness. This has relevance to fitness to drive assessments in OSA, and the lack of controls did not impact our ability to address this important question.

Bedtime and wake time and all data collection during the extended wakefulness period were standardised in this laboratory study, and circadian phase measurements were not conducted. Thus, potential circadian effects on the measured variables could not be examined and warrant further investigation. Also, the impact of OSA treatment on wake EEG and vigilance performance, particularly targeting vulnerable patients, is warranted. Strengths of this study include the larger sample size relative to previous studies and a highly characterised clinical sample of patients regarding objective sleep and clinical evaluation, quantitative EEG and vigilance/driving assessments.

### 5.1 Interpretation

We have shown that EEG theta and delta power during rested wake using a simple standardised test (KDT) was significantly elevated in patients with OSA who exhibited significant PVT and driving performance impairment following extended wakefulness. This highlights the potential clinical utility of using resting wake EEG assessments in clinical practice to complement current questionnaire, PSG and MWT/MSLT testing, to gain additional prognostic insight into which patients with OSA may be more vulnerable to sleepiness‐related vigilance impairment and driving risk. Before clinical use, it would be important to establish independent validation of these findings, establish thresholds for classifying at‐risk patients, and assess other potential congruent markers of vigilance impairment. Importantly, it will ultimately be necessary to determine if these wake EEG markers prove useful in predicting real‐world driving impairment and accident risk.

## Author Contributions

**Andrew Vakulin:** conceptualization, investigation, methodology, writing – review and editing, formal analysis, data curation, supervision, visualization, software. **Garry Cho:** writing – original draft, methodology, writing – review and editing. **David Stevens:** investigation, writing – review and editing. **Nathaniel S. Marshall:** writing – review and editing, methodology, supervision. **Hannah Openshaw:** writing – review and editing, investigation. **Delwyn J. Bartlett:** investigation, writing – review and editing. **Caroline D. Rae:** investigation, writing – review and editing. **Keith K. H. Wong:** investigation, writing – review and editing. **R. Doug McEvoy:** investigation, writing – review and editing, supervision. **Ronald R. Grunstein:** conceptualization, investigation, funding acquisition, methodology, writing – review and editing, supervision, resources, project administration. **Angela L. D'Rozario:** conceptualization, investigation, funding acquisition, writing – review and editing, methodology, resources.

## Conflicts of Interest

This study was supported by an NHMRC grant (GNT1028624). A.V. was supported by an NHMRC Early Career Research Fellowship. A.L.D. was supported by a NHMRC‐ARC Dementia Research Development Fellowship (1107716), R.D.M. was supported by NHMRC Research Practitioner Fellowships. C.D.R. reports grants from National Health and Medical Research Council (Australia), during the conduct of the study; personal fees from Springer, personal fees from Elsevier, outside the submitted work. R.R.G. was supported by a NHMRC Senior Principal Research Fellowship (1106974). A.V. has received competitive research funding and equipment from ResMed and Philips Respironics for research unrelated to and outside the submitted work. R.D.M. has received research funding and equipment support from Philips Respironics, ResMed and Fisher and Paykel for research unrelated to and outside the submitted work. R.R.G.'s department has received support from Philips, Avadel, Nyxoah, Merck, Eisai, Eli Lilly unrelated to this work. Other authors have no further disclosures.

## Supporting information


**Figure S1.** Photographs depicting the AusEd driving simulator set‐up in the sleep laboratory.

**Figure S2.** Shows the summary of the Two‐Step Cluster Analysis output used to define the vulnerable vs resistant OSA drivers based on their PVT performance (averaged from 3 consecutive early morning tests at 20, 22 and 24h of wakefulness) and the 2nd driving simulator test (at 22.5h of wakefulness) representing circadian nadir and the worse time point for driving and vigilance performance. Figure S2A shows that there were 2 clear clusters that resulted from the 4 data inputs and this process was automatic and unsupervised. The log‐likelihood distance measure and Bayesian (BIC) clustering criteria was applied and the Silhouette measure of cohesion and separation of the 2 clusters was 0.6 which corresponds to Good cluster quality. Figure S2B shows the size of the 2 clusters with 16 OSA patients (29.6%) defined as vulnerable and 38 (70.4%) defined as resistant.

**Figure S3.** Shows a direct comparison between the vulnerable vs. resistant clusters across the four data inputs used in the model. It is clear that relative to the resistant group in blue, the vulnerable group (red) exhibited significantly more frequent PVT lapses, slower PVT Reciprocal Reaction Time (PVT RRT), more frequent driving simulator crashes and greater steering deviations.

**Data S1.** Supporting Information.

## Data Availability

The data that support the findings of this study are available on request from the corresponding author. The data are not publicly available due to privacy or ethical restrictions.
