# Supplementary figures and images for: Wake EEG and Sleep Hypoxemia Predicts Poor Driving and Vigilance Following Extended Wakefulness in People With OSA
Source: J Sleep Res. 2025 Jul 9;35(1):e70131. doi: 10.1111/jsr.70131 (PMC12856125; doi:10.1111/jsr.70131)

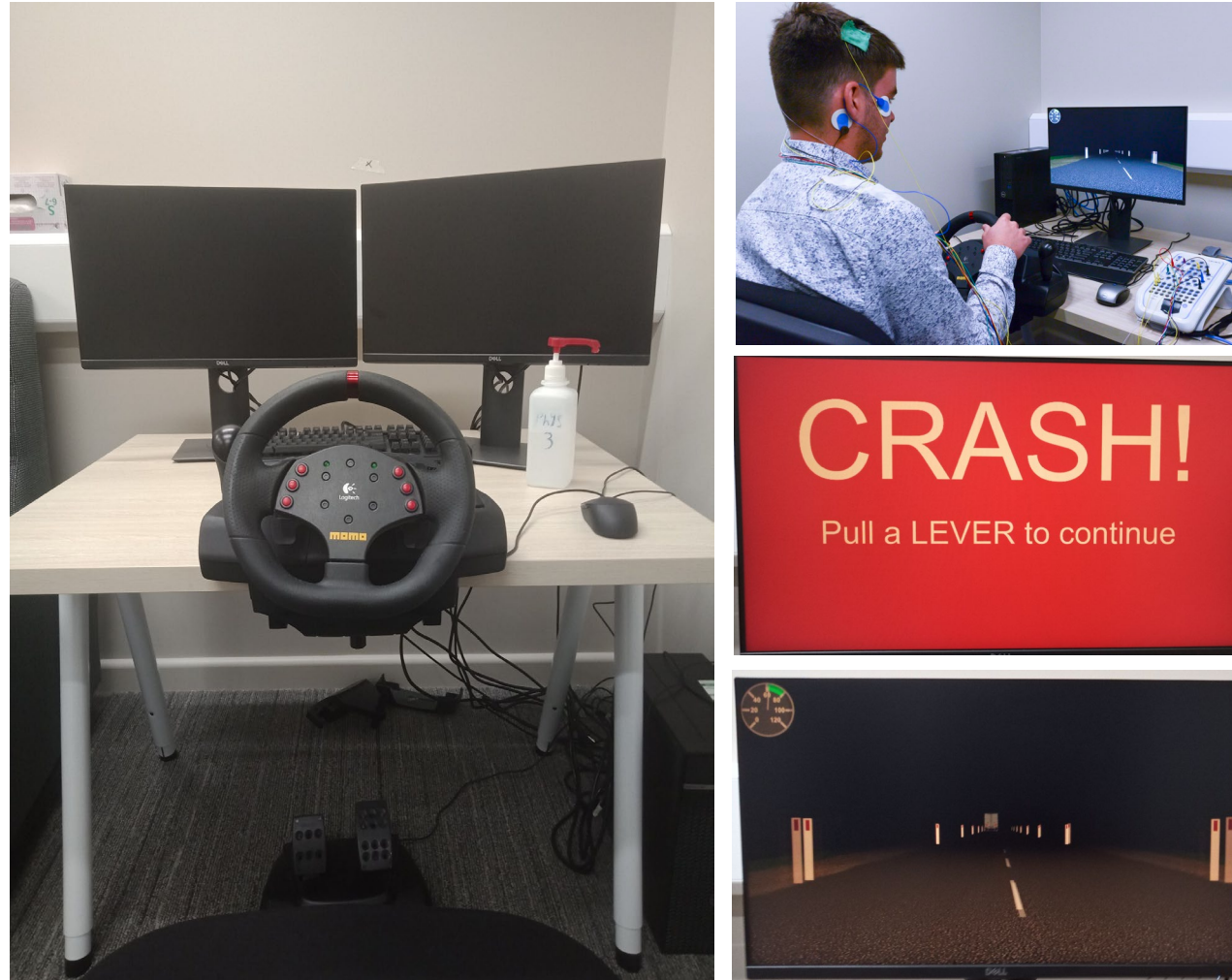

**Figure S1:** Photographs depicting the AusEd driving simulator set-up in the sleep laboratory.

Supplement: Supplementary file 1 — Figure S1. Photographs depicting the AusEd driving simulator set‐up in the sleep laboratory. [file JSR-35-e70131-s003.pdf]
