# Supplementary material for: Wake EEG and Sleep Hypoxemia Predicts Poor Driving and Vigilance Following Extended Wakefulness in People With OSA
Source: J Sleep Res. 2025 Jul 9;35(1):e70131. doi: 10.1111/jsr.70131 (PMC12856125; doi:10.1111/jsr.70131)
Supplement: Supplementary file 2 — Figure S2. Shows the summary of the Two‐Step Cluster Analysis output used to define the vulnerable vs resistant OSA drivers based on their PVT performance (averaged from 3 consecutive early morning tests at 20, 22 and 24h of wakefulness) and the 2nd driving simulator test (at 22.5h of wakefulness) representing circadian nadir and the worse time point for driving and vigilance performance. Figure S2A shows that there were 2 clear clusters that resulted from the 4 data inputs and this process was automatic and unsupervised. The log‐likelihood distance measure and Bayesian (BIC) clustering criteria was applied and the Silhouette measure of cohesion and separation of the 2 clusters was 0.6 which corresponds to Good cluster quality. Figure S2B shows the size of the 2 clusters with 16 OSA patients (29.6%) defined as vulnerable and 38 (70.4%) defined as resistant. [file JSR-35-e70131-s002.pdf]

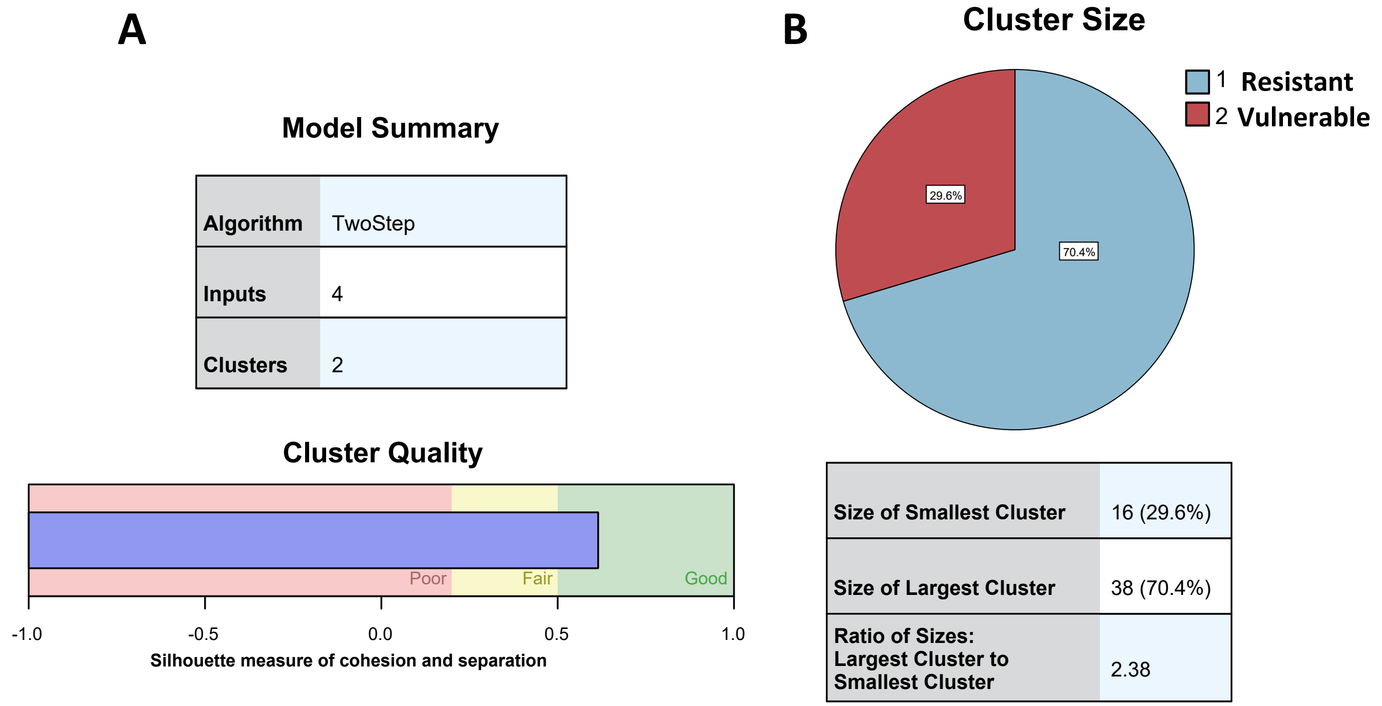

Figure S2: Shows the summary of the Two-Step Cluster Analysis output used to define the vulnerable vs resistant OSA drivers based on their PVT performance (averaged from 3 consecutive early morning tests at 20, 22 and 24hrs of wakefulness) and the 2nd driving simulator test (at 22.5hrs of wakefulness) representing circadian nadir and the worse time point for driving and vigilance performance. Figure S2A shows that there were 2 clear clusters that resulted from the 4 data inputs and this process was automatic and unsupervised. The log-likelihood distance measure and Bayesian (BIC) clustering criteria was applied and the Silhouette measure of cohesion and separation of the 2 clusters was 0.6 which corresponds to Good cluster quality. Figure S2B shows the size of the 2 clusters with 16 OSA patients (29.6%) defined as vulnerable and 38 (70.4%) defined as resistant.
