# Supplementary material for: Wake EEG and Sleep Hypoxemia Predicts Poor Driving and Vigilance Following Extended Wakefulness in People With OSA
Source: J Sleep Res. 2025 Jul 9;35(1):e70131. doi: 10.1111/jsr.70131 (PMC12856125; doi:10.1111/jsr.70131)
Supplement: Supplementary file 3 — Figure S3. Shows a direct comparison between the vulnerable vs. resistant clusters across the four data inputs used in the model. It is clear that relative to the resistant group in blue, the vulnerable group (red) exhibited significantly more frequent PVT lapses, slower PVT Reciprocal Reaction Time (PVT RRT), more frequent driving simulator crashes and greater steering deviations. [file JSR-35-e70131-s001.pdf]

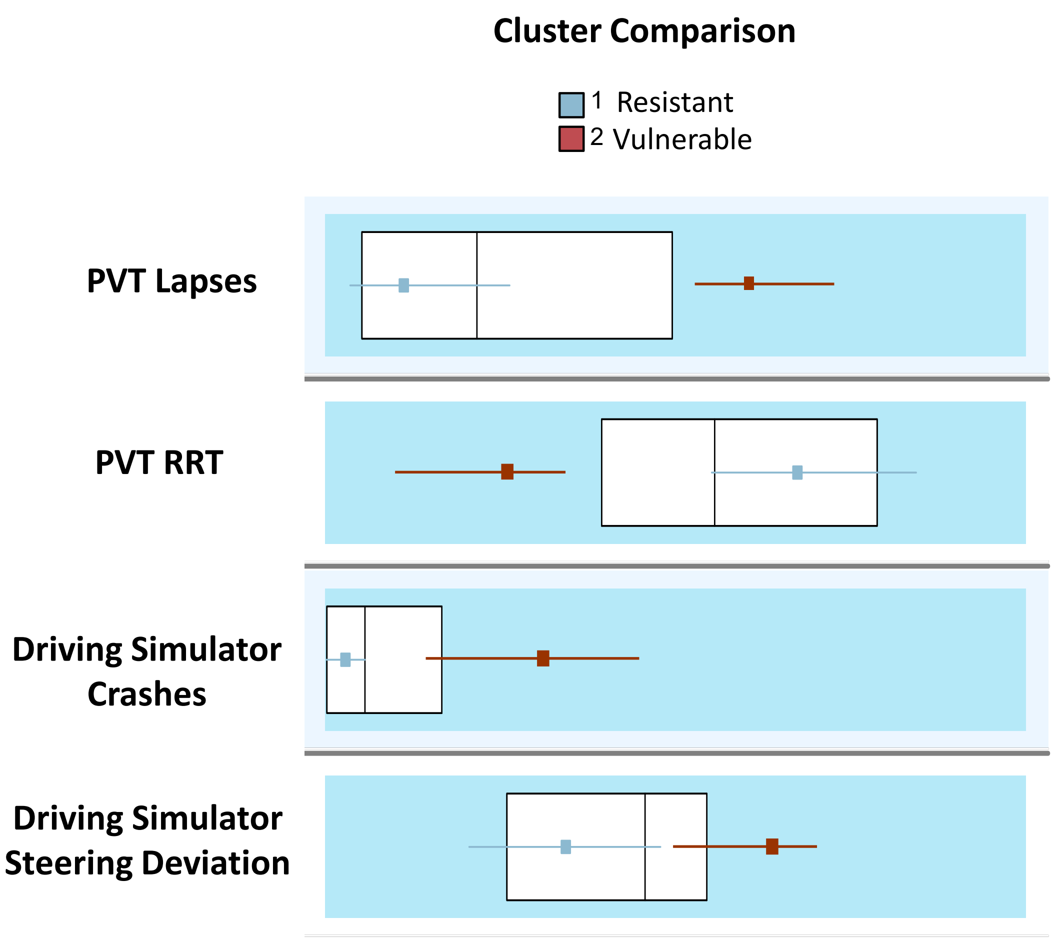

Figure S3: Shows a direct comparison between the vulnerable vs resistant clusters across the 4 data inputs used in the model. It is clear that relative to the resistant group in blue, the vulnerable group (red) exhibited significantly more frequent PVT lapses, slower PVT Reciprocal Reaction Time (PVT RRT), more frequent driving simulator crashes and greater steering deviations.
