# Supplementary material for: Wake EEG and Sleep Hypoxemia Predicts Poor Driving and Vigilance Following Extended Wakefulness in People With OSA
Source: J Sleep Res. 2025 Jul 9;35(1):e70131. doi: 10.1111/jsr.70131 (PMC12856125; doi:10.1111/jsr.70131)
Supplement: Supplementary file 4 — Data S1. Supporting Information. [file JSR-35-e70131-s004.docx]

**SUPPLEMENTARY MATERIAL**

**Wake EEG and sleep hypoxemia predicts poor driving and vigilance following extended wakefulness in people with OSA**

**Authors:** *****Andrew Vakulin^1,2^, *Garry Cho^2,^ David Stevens^1,3^, Nathaniel S. Marshall^2,4^, Hannah Openshaw^2^, Delwyn J. Bartlett^,2,4^, Caroline D. Rae^5,6^, Keith K. H. Wong^,2,4,8^, R. Doug McEvoy^1^, # Ronald R. Grunstein^2,4,8^, #Angela L. D’Rozario^2,7^

* co-first authors, # co-senior authors.

-first authors, # co-senior authors.

1. Flinders Health and Medical Research Institute, Sleep Health / Adelaide Institute for Sleep Health, College of Medicine and Public Health, Flinders University, Adelaide, Australia;
2. Sleep and Circadian Research Group (CIRUS), Woolcock Institute of Medical Research, Sydney, Australia;
3. Centre for Nutrition and Gastrointestinal Diseases, South Australian Health & Medical Research Institute, Adelaide, Australia;
4. Faculty of Medicine and Health, University of Sydney, Sydney, Australia
5. Neuroscience Research Australia, Sydney, Australia.
6. School of Psychology, The University of New South Wales, Sydney, Australia.
7. School of Psychology, Faculty of Science, Brain and Mind Centre and Charles Perkins Centre, University of Sydney, Sydney, Australia.
8. Royal Prince Alfred Hospital, and Sydney Health Partners, Sydney, Australia;

**Additional Methodology Information**

***AusEd Driving Simulator assessment***

The AusEd driving simulator task (Woolcock Institute of Medical Research, Sydney, Australia) ^1^ simulates driving on a country road at night. The driving task was performed on a desktop computer with a Logitech MOMO steering wheel and pedals for acceleration and braking and involved a 90-minutes monotonous country driving scenario as described previously ^2,3^. In brief, steering deviation was measured from the average deviation in centimeters from the driver’s median lane position (each lane was 360cm wide) sampled at 30Hz. Participants were instructed to maintain speed within 60-80 km/h, but to apply the brakes as quickly as possible whenever a slow moving truck was presented ahead in the driving lane.

***Backward Stepwise Regression***

Backward stepwise multiple linear regression models were used to examine which baseline demographic, PSG and baseline wake qEEG variables were significant predictors of continuous PVT and driving simulator performance following extended wakefulness. For the purpose of this analysis the three PVT and driving performance variables from the 2^nd^ driving test during the extended wake period were reduced to a single Vigilance Performance Factor using principal component factor analysis. We considered the absolute vigilance performance (PVT and driving simulator performance) following extended wakefulness as the primary performance outcome of interest when sleepiness related impairment is expected to be highest, as this is more relevant and interpretable clinically, rather than examining the change in performance from baseline to extended wake. We therefore did not attempt to adjust for baseline PVT and driving performance in our extended wakefulness regression models or when classifying patients into vulnerability groups. The baseline wake EEG was quantified as the average wake EEG delta, theta, alpha and beta power averaged from three consecutive KDT tests at 8, 10 and 12-hours of wakefulness, which was considered to represent a rested baseline period of the protocol. Criteria for predictor selection were from those that 1) demonstrated significant correlations with at least one measure of PVT and driving simulator performance at p<0.01, and 2) standard measures considered clinically important. Model criteria for keeping a predictor variable in the model were set at p<0.01 to account for a relatively small sample size. All final models included baseline variables age, BMI, ESS, total sleep time, AHI, sleep onset latency, O2 Nadir and wake EEG variable of interest. We also performed additional sensitivity analysis to examine if alternate metrics of hypoxemia, the ODI and average oxygen desaturation (which correlated with performance outcomes), were predictive of vigilance outcome in regression models.

**Supplementary Figure Legends**

**Figure S1:** Photographs depicting the AusEd driving simulator set-up in the sleep laboratory.

**Figure S2:** Shows the summary of the Two-Step Cluster Analysis output used to define the vulnerable vs resistant OSA drivers based on their PVT performance (averaged from 3 consecutive early morning tests at 20, 22 and 24hrs of wakefulness) and the 2^nd^ driving simulator test (at 22.5hrs of wakefulness) representing circadian nadir and the worse time point for driving and vigilance performance. Figure S2A shows that there were 2 clear clusters that resulted from the 4 data inputs and this process was automatic and unsupervised. The log-likelihood distance measure and Bayesian (BIC) clustering criteria was applied and the Silhouette measure of cohesion and separation of the 2 clusters was 0.6 which corresponds to Good cluster quality. Figure S2B shows the size of the 2 clusters with 16 OSA patients (29.6%) defined as vulnerable and 38 (70.4%) defined as resistant.

**Figure S3**: Shows a direct comparison between the vulnerable vs resistant clusters across the 4 data inputs used in the model. It is clear that relative to the resistant group in blue, the vulnerable group (red) exhibited significantly more frequent PVT lapses, slower PVT Reciprocal Reaction Time (PVT RRT), more frequent driving simulator crashes and greater steering deviations.

**References**

1. Desai AV, Wilsmore B, Bartlett DJ, et al. The utility of the AusEd driving simulator in the clinical assessment of driver fatigue. *Behav Res Methods.* 2007;39(3):673-681.

2. Vakulin A, Catcheside PG, Baulk SD, et al. Individual variability and predictors of driving simulator impairment in patients with obstructive sleep apnea. *J Clin Sleep Med.* 2014;10(6):647-655.

3. Vakulin A, Green MA, D'Rozario AL, et al. Brain mitochondrial dysfunction and driving simulator performance in untreated obstructive sleep apnea. *J Sleep Res.* 2021:e13482.
